# Newborn Screening in Fabry Disease

**DOI:** 10.3390/ijms262412125

**Published:** 2025-12-17

**Authors:** Marta Olszewska, Krzysztof Schwermer, Krzysztof Pawlaczyk

**Affiliations:** Department of Nephrology, Transplantology and Internal Diseases, Poznan University of Medical Sciences, 60-355 Poznan, Poland; marta.olszewska@usk.poznan.pl (M.O.); krzysztof.schwermer@usk.poznan.pl (K.S.)

**Keywords:** Fabry disease, lysosomal disease, newborn screening

## Abstract

Fabry disease (FD) is an X-linked genetic disease caused by deficient α galactosidase A activity, leading to a lysosomal storage disorder of globotriaosylceramide, causing organ damages. There are two most common clinical manifestations of the disease: classic FD with a typical onset of symptoms in childhood in males, and later-onset variants which may include female heterozygotes. The highly heterogeneous and nonspecific nature of FD’s symptoms and limited physicians’ awareness result in a significant diagnostic and therapeutic delay. Even though the implementation of newborn screening (NBS) gives us an opportunity for early diagnosis and timely treatment, it has not yet been universally adopted. Over twenty pilot studies and screening programs worldwide have been published, showing that FD is more prevalent than previously estimated, exceeding 1 in 40,000 males, mainly due to the high incidence of variants of unknown significance (VUSs). They also raised controversies regarding the diagnostic methods, results interpretation, ethical issues, clinical approach, and economic burden. This review analyzes recent studies of NBS for FD; examines the screening methods, prevalence findings, and natural history data; and assesses the benefits and risks of NBS. We conclude with suggestions for the screening program design and research priorities to ensure that screening leads to improved health outcomes with acceptable costs and psychosocial impact.

## 1. Introduction

Fabry disease is a rare lysosomal storage disorder caused by a deficient or markedly reduced activity of the lysosomal enzyme α-galactosidase A (α-Gal A), encoded by the GLA gene located on the X chromosome [1].

The disease was first described in 1898 by two physicians—William Anderson from St Thomas’ Hospital in London and Johannes Fabry from the University of Bonn—working independently of each other. In 1967, Roscoe Brady found out that Fabry disease is caused by a deficiency of enzyme activity [2], and, in 1970, the enzyme was identified as α-galactosidase A [3]. Later, in 1985, the genetic background of Fabry disease was confirmed by Robert J. Desnick’s group by discovering a mutation in the GLA gene [4]. The enzymatic defect results in the progressive accumulation of Gb3 and its deacylated derivative lyso-Gb3, both of which trigger inflammatory pathways and result in irreversible cellular damage, multiorgan failure, and premature death. To date, more than 1000 pathogenic GLA gene variants have been described, although a substantial number still remains classified as variants of uncertain significance (VUSs).

**Figure 1:**
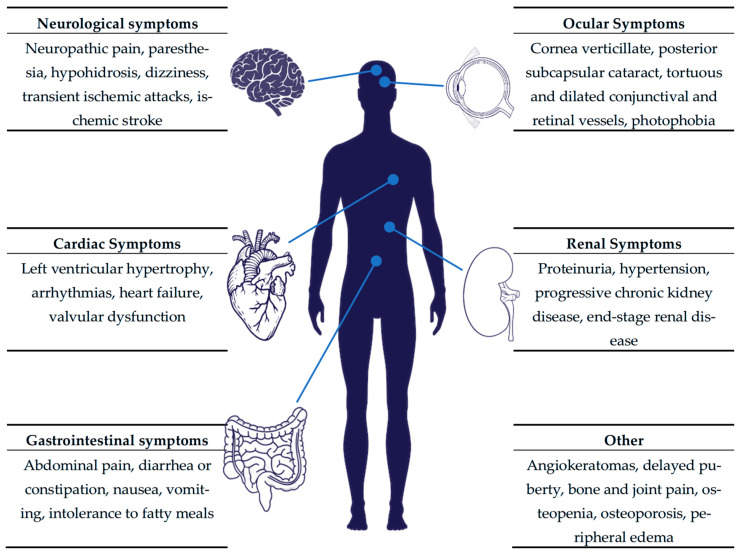
Symptoms and organ involvement in Fabry disease.

The clinical phenotype of Fabry disease is highly heterogeneous. Individuals with the classic phenotype typically develop symptoms in childhood, including angiokeratomas, neuropathic pain, hypohidrosis (i.e., decreased sweating), hearing loss, and gastrointestinal disturbances [5,6]. Without a timely diagnosis and treatment, the progressive involvement of the kidneys, heart, and both the central (cerebrovascular disease) and peripheral nervous systems follows. Patients with later-onset Fabry disease are usually diagnosed in adulthood, exhibiting a variable degree of cardiac, renal, and neurological involvement [7]. The broad symptomatic spectrum of Fabry disease is presented in Figure 1. Previously, heterozygous females were considered asymptomatic carriers responsible for transmitting the pathogenic variant to their offspring. However, long-term clinical observations have demonstrated that, unlike other X-linked disorders, these females do frequently develop clinical manifestations. This variability is attributed to random X-chromosome inactivation, which leads to a broad phenotypic spectrum [8].

In males with the classic phenotype, significantly reduced α-Gal A activity measured in dried blood spots (DBSs), leukocytes, plasma, or fibroblasts can confirm the diagnosis. In contrast, the enzyme activity in females and in later-onset male patients may fall within the normal range; thus, a molecular genetic analysis of the GLA gene is required for a definitive diagnosis in these groups [9].

The broad clinical variability and nonspecific nature of symptoms, combined with limited physicians’ awareness, often result in a significant diagnostic delay. Data from the Fabry Outcome Survey (FOS) indicate a mean delay between symptom onset and diagnosis of 13.7 years in males and 16.3 years in females [10]. Moreover, in an analysis of 366 FOS participants, more than 25% were initially misdiagnosed. The same dataset demonstrates that untreated Fabry disease reduces life expectancy by approximately 20 years in men and 15 years in women [10].

Early therapeutic intervention, particularly enzyme replacement therapy, is essential for preventing or attenuating irreversible organ damage [11], whereas the delayed initiation of treatment is associated with diminished efficacy [12]. Enzyme replacement therapy was introduced at the beginning of the 21st century by Christine Eng et al. [13] and Raphael Schiffmann et al. [14], and, currently, two therapeutic approaches are available: the intravenous administration of recombinant human α-Gal A (agalsidase alfa, 0.2 mg/kg, or agalsidase beta, 1 mg/kg) [13], and oral chaperone therapy with migalastat, which is indicated for patients harboring amenable GLA mutations [15].

Even though the implementation of newborn screening (NBS) for Fabry disease offers a great opportunity for early diagnosis and the timely initiation of therapy, such screening has not yet been universally adopted. In the following review, we summarize the current global status of newborn screening for Fabry disease and discuss its benefits and limitations.

## 2. Screening Methods

The diagnostic approach to Fabry disease typically consists of a two-tiered strategy, which includes an initial biochemical screening test and subsequent genetic confirmation. The screening phase involves the measurement of α-Gal A enzymatic activity, whereas the molecular analysis of the GLA gene is used to confirm or exclude the diagnosis.

### 2.1. First Tier—Enzyme Activity Assessment

Enzymatic screening is most commonly performed using DBS samples, which facilitate large-scale testing and sample transport. A broad range of analytical methods has been developed for DBS-based assays, including fluorometric measurement, immunoquantification, digital microfluidics (DMF), and tandem mass spectrometry (MS/MS). Among these, DMF and MS/MS are currently the most widely adopted due to their high analytical throughput and precision.

The baseline enzyme activity might be affected by many factors, e.g., newborn hematocrit, blood-spot volume, time between collection and assay, ambient temperature during drying/transport, and seasonal effects. When the α-Gal A activity falls below the established cut-off threshold, the retesting of the same or a new sample is recommended to exclude preanalytical variability. The prevalence of pseudodeficiency or certain benign GLA variants may lower the enzyme activity without a clinical disease—in these cases, the cut-off setting and second-tier tests must be performed. Persistently reduced enzyme activity indicates a high likelihood of Fabry disease and indicates the necessity for further genetic evaluation. Although techniques such as MS/MS, DMF, and immunocapture assays demonstrate excellent sensitivity, their specificity remains suboptimal and should be an area of ongoing refinement [16]. Due to certain diagnostic methods’ significant differences in sensitivity and specificity in male and female patients, sex-specific testing algorithms should be advised [17]. It is important to note that enzymatic testing is not reliable for identifying heterozygous females, as random X-chromosome inactivation may result in normal or near-normal α-Gal A activity despite the presence of pathogenic GLA gene variants. Moreover, different laboratories use different methods, like fluorometric assays, digital microfluidics, or tandem-MS/MS and, while the enzyme activity results are method-dependent, it is impossible to set a numeric cut-off for all platforms. Therefore, setting a proper cut-off might be essential for a proper diagnosis: increasing the cut-off improves sensitivity, especially for detecting females with mildly reduced activity, but it lowers the specificity and increases the detection of VUSs and benign mutations. Consequently, genetic testing remains the preferred screening and diagnostic method in women with a clinical suspicion or family history suggestive of Fabry disease. Classic Fabry males typically have extremely low α-Gal A activity; thus, lowering the cut-off will mainly impact the detection of borderline and later-onset males. Moreover, not all males with low α-Gal A activity were confirmed to have Fabry disease, which also indicates the need for confirmatory testing with tools such as gene sequencing and plasma lyso-Gb3.

### 2.2. Second-Tier Diagnostics—Measurement of Biomarkers

An assessment of the serum or urinary concentrations of globotriaosylsphingosine (lyso-Gb3) can enhance the specificity of Fabry disease screening, and give additional information when the gene sequencing results are negative or inconclusive and help identify patients with classic and late-onset phenotypes [18]. Elevated lyso-Gb3 levels have shown to possess both diagnostic and prognostic value, correlating with the disease phenotype and clinical severity in both males and females [19,20]. Moreover, lyso-Gb3 concentrations can serve as a useful biomarker for disease monitoring and therapeutic response. In the neonatal period, markedly increased lyso-Gb3 levels are highly suggestive of classic Fabry disease [21]; however, normal concentrations do not exclude the possibility of later-onset variants [22]. The levels of lyso-GB3 are more suitable for determining males with Fabry disease as they are usually significantly higher in males than in females. The analysis of data from The Lantern Project showed that the level of lyso-Gb3 correlates with the type of GLA mutation; all females with loss-of-function (LOF) variants had elevated lyso-Gb3 levels, whereas female carriers of missense GLA variants had variable lyso-Gb3 levels. In 16 female patients, pathogenic (P)/likely pathogenic (LP) missense variants [including c.1087C>T (p.R363C), c.1088G>A (p.R363H), c.593T>C (p.I198T), c.644A>G (p.N215S), c.335G>A (p.R112H), c.337T>C (p.F113L), and c.835C>G (p.Q279E)] were identified with normal lyso-Gb3 levels [23]. The proposed lyso-Gb3 cut-off values may help distinguish classic from later-onset males, although no universally validated neonatal thresholds currently exist. Hence, the interpretation of lyso-Gb3 results should always be integrated with enzymatic and genetic findings to ensure accurate diagnosis and disease classification.

### 2.3. Genetic Screening

The sequencing of the GLA gene, located on the X chromosome, represents the ultimate diagnostic method for confirming Fabry disease. A molecular analysis enables the precise identification of the underlying variant, allowing the differentiation between classic and late-onset forms, as well as the identification of benign or nonpathogenic variants. Furthermore, GLA sequencing is essential for detecting heterozygous female carriers, in whom enzymatic testing alone may bring inconclusive results. Although molecular assays for the NBS of Fabry disease have been investigated, they remain under development and are not yet suitable for large-scale application. For example, the high-resolution melting method can analyze extensive regions covering all seven exons of the GLA gene, but it demonstrates limited sensitivity and suboptimal reliability in males [24]. Similarly, the Agena iPLEX platform detects only known pathogenic variants [25], which restricts its utility for comprehensive screening. Nevertheless, continuing advances in sequencing technology may soon bestow molecular assays a practical component of early diagnostic workflows.

Some screening programs are now considering the direct addition of molecular testing (through genotyping or sequencing) as part of the initial screening tier. Such an approach could enhance the detection rate of disease-causing variants but simultaneously introduces interpretative challenges, particularly in relation to VUSs and those with an unclear pathogenic potential [26].

Table 1 shows the methods used in NBS for FD.

## 3. Managing Positive Neonates

In males, markedly reduced α-Gal A activity alongside pathogenic GLA variants confirms FD. In females, due to possible random X-chromosome inactivation leading to variable enzyme levels, genetic testing remains the definitive diagnostic method. Upon confirmation of Fabry disease, a comprehensive baseline clinical assessment should be performed to define the extent of organ involvement. This evaluation should comprise electrocardiography (ECG) and echocardiography, to estimate cardiac morphology and function; the assessment of renal function by the measurement of the estimated glomerular filtration rate (eGFR) and urinary protein excretion, and a renal ultrasound, which can reveal parapelvic cysts or enlarged kidneys in the early stages due to sphingolipids accumulation [27]; and an ophthalmologic examination to identify cornea verticillata or retinal vessel tortuosity [28]. Additional evaluations, for example, a brain MRI [29,30] and audiologic testing [31], should be advised in specific cases to detect the subclinical involvement of the central nervous system and hearing apparatus. These investigations establish the initial disease profile and guide subsequent clinical management.

The frequency of follow-up depends on the clinical phenotype. Infants with the classic phenotype should undergo multidisciplinary evaluations every six months to screen for early organ involvement. In contrast, for patients with atypical or later-onset variants, Gragnaniello et al. recommend annual evaluations using the same diagnostic modalities [32]. Nevertheless, determining evidence-based algorithms for surveillance and the timely initiation of disease-specific therapy, particularly in patients with unclassified variants, VUSs, or later-onset forms, have changed over the years and still remains a critical unmet need. In 2006, an international panel of experts developed guidelines for the therapeutic management of Fabry disease, which stated that ERT should be initiated in pediatric males at the time of the development of significant symptoms such as chronic acroparesthesia resistant to conventional therapy, persistent proteinuria (>300 mg/24 h), a glomerular filtration rate < 80 mL/min/1.73 m^2^, clinically significant cardiac involvement, a previous cerebrovascular accident, or a history of transient ischaemic attacks or ischaemic changes on brain magnetic resonance imaging. In asymptomatic patients, therapy should be considered at 10–13 years of age [33,34]. The European Fabry Working Group achieved consensus that treatment with ERT may be considered in males with classical FD of 16 years or older even if they have no symptoms or clinical signs of organ involvement [35]. The 2019 French consensus recommendations for the treatment of pediatric patients indicate that ERT should be considered for asymptomatic boys from the age of 7 years as the initiation of treatment in childhood may prevent organ damage in later life [36]. Long-term observational studies are essential in order to improve our understanding of the natural history of the disease, refine the risk stratification, optimize the treatment timing, and enhance the outcomes for all patients with Fabry disease.

## 4. Epidemiological Insights from Population Studies

In Northeastern Italy, a total of 44,411 newborns were tested for Fabry disease over a five-year period via α-Gal A enzyme activity in DBSs. The study reveals a higher incidence of the disease (≈1 in 7879 newborns) than was previously estimated. It also confirms lyso-Gb3 as a useful biomarker for monitoring; however, it remains unreliable as a second-tier screening test [32].

In Taiwan, the prevalence of Fabry disease has been reported to be remarkably high. In a population-based screening study involving approximately 90,000 male newborns, the frequency of GLA mutations was estimated at about 1 in 1250 males. Notably, 82% of identified carriers harbored the intronic variant IVS4+919G>A, which is associated primarily with the cardiac variant of Fabry disease. However, only a group of individuals carrying this mutation develops overt clinical manifestations [37].

In Japan, a pilot NBS program conducted in Fukuoka evaluated 21,170 infants for α-Gal A activity. Several newborns were found to have reduced enzymatic activity, and pathogenic GLA variants were confirmed in three of them, corresponding with an estimated frequency of 1 in 7057 male births. Additional VUSs were also identified, further underlining the ongoing challenge of interpreting genetic findings in large-scale screening initiatives [38].

In China, a NBS study conducted in Nanjing examined 17,171 infants and reported a prevalence of pathogenic GLA variants of about 1 in 1321 males. The majority of identified mutations were region-specific, reflecting the local genetic variation within the population. The residual α-Gal A activity varied considerably among affected individuals, indicating a heterogeneity in enzymatic expression and potential differences in disease phenotype [26].

It is difficult to compare the studies presented above, because they differ in many aspects, such as screening techniques and the classification of later-onset, benign variants and VUSs. Each demonstrates that FD is considerably more prevalent than previously estimated, far exceeding the earlier projection of approximately 1 in 40,000 males reported by Desnick et al. [39], ranging from ~1:1250 males in Taiwan to 1:6000–1:10,000 in European and Japanese studies. The enzyme-only first tier (DBS α-Gal A) may miss many heterozygous females, while programs with GLA sequencing detect more females but also increase VUS detection. Some of these studies included any pathogenic or likely pathogenic GLA variants found by sequencing, increasing the prevalence, while, in many cases, the affected infants remain asymptomatic for years, causing major challenges in determining the optimal timing of therapeutic intervention. Other NBS pilots counted only classical variants, resulting in a lower disease prevalence. The other driver of variability is population genetics—founder alleles will show a much higher NBS prevalence (e.g., there is a high prevalence in Taiwan’s population caused by a common later-onset founder allele (IVS4+919G>A), while other regions without such alleles show lower yields). These findings highlight the limitations of the currently available laboratory methodologies and emphasize the demand for continued improvement to achieve diagnostic tools with both a high sensitivity and specificity [40].

In Poland, a national NBS program for approximately 30 diseases is a standard of care procedure since 1994; however, FD has not been yet included in the diagnostic panel. Patients’ organizations (e.g., National Forum for Rare Disease Therapy) and most Fabry experts campaign for extending the NBS panel to lysosomal storage diseases, providing health improvement (early diagnosis, and prompt treatment before the development of complications) and potential savings as the benefit. On the other hand, the Agency for Health Technology Assessment and Tariff System is very cautious and indicates a high incidence of VUSs, and later-onset or benign variants in other screening programs, a lack of clinical practice guidelines, and economic burden. Nevertheless, in 2025, in Poland, pilot extended NBS programs, including Pompe, Fabry, and Gaucher diseases, as well as MPS I, are planned in several voivodeships, with hopes of being implemented permanently.

## 5. Benefits of Newborn Screening

In 1968, Wilson and Jungner described 10 principles for evaluating public health screening programs, which should be met prior to introduction: disease importance, natural history, acceptable test, treatment, facilities, agreed policy, cost-effectiveness, and continuing process. These criteria have been updated to accommodate modern public health problems, such as ethic and legal issues, economic feasibility, and modern treatment.

As mentioned above, the diagnosis of Fabry disease is associated with a 13.7-year delay in males and a 16.3-year delay in females, respectively. Because the symptoms may be subtle or nonspecific, a significant number of patients remain misdiagnosed or undiagnosed for many years [41] or have to experience a ‘diagnostic odyssey’ [42] before proper diagnosis. The primary objective of implementing NBS for Fabry disease is, therefore, the early identification of affected infants with the classic phenotype and the prompt initiation of enzyme replacement therapy (ERT) or other disease-specific treatment. NBS enables the structured longitudinal monitoring of biomarkers such as plasma or urinary globotriaosylsphingosine (lyso-Gb3), subclinical organ changes, and the onset of clinical symptoms, supporting timely therapeutic decision-making. This approach has been confirmed to slow the disease progression and preserve organ function, particularly in the kidneys, heart, and central nervous system [1].

However, not only does NBS capture classic forms, but, additionally, it detects individuals with GLA gene variants associated with the late-onset phenotype or VUSs who may remain asymptomatic for years. This enables longitudinal monitoring, the early recognition of organ involvement, and the possibility of early therapeutic intervention when necessary. Moreover, these follow-up studies contribute to the refinement of genotype–phenotype correlations and the better understanding of disease heterogeneity. Confirming a pathogenic GLA variant in a newborn facilitates cascade screening, allowing for the identification of previously undiagnosed carriers or affected relatives, for example, heterozygous females who may later develop symptoms. Family (cascade) screening extends the diagnostic process on other members of the family and stands as a grave component of FD case detection strategies. Since FD is inherited in an X-linked manner, the identification of one affected member often leads to other at-risk relatives being identified across generations. Previous studies have shown that most infants identified through NBS had no previously recognized family history of Fabry disease [32]. A review of the literature on cascade family screening demonstrated that, among 365 index cases, an additional 1744 affected relatives were consequently identified [43,44]. These findings highlight the diagnostic yield and public health value of systematic family-based genetic testing in lysosomal storage disorders. Early identification also provides families with access to genetic counseling, reproductive advice, and psychosocial support. Moreover, the introduction of universal NBS ensures equity and fairness in the access to diagnostic and therapeutic resources, regardless of socioeconomic status or geographic location.

The pursuit of developing NBS programs has contributed to significant advancements in laboratory and screening technologies such as high-throughput enzymatic assays, digital microfluidics, and tandem mass spectrometry (MS/MS), while multi-tier protocols, consisting of an α-galactosidase A enzyme activity measurement with lyso-Gb3 quantification and confirmatory GLA sequencing, have improved the precision and minimized the false positive results. These methods are now feasible at a population scale. Furthermore, NBS programs give us new epidemiological insight, challenging the perception of FD as an ultra-rare disorder. Fabry disease, with a previously estimated prevalence ranging from 1 in 40,000 to 1 in 170,000 live births [45], is likely more common than previously thought. Pilot screening programs in Italy, Taiwan, and Japan have reported incidence rates ranging from 1:3100 to 1:8800 male newborns. These data further magnify the impact of the screening program. By identifying affected newborns, NBS provides a cohort for future studies and contributes to the research on therapies such as chaperone therapy, substrate reduction, mRNA, and gene therapy trials. These novel agents might lead to new therapeutic strategies. NBS enables a better understanding of the natural course of the disease and genotype–phenotype correlations. Although the data are still being accumulated, they suggest that early identification followed by timely therapy improve quality of life and extend survival. Finally, by preventing the development of disease complications, NBS might prove cost-effective at the population level.

## 6. Challenges of Newborn Screening

While the benefits of early detection and treatment in individuals with the classic form of Fabry disease are well-established, it is important to recognize that the implementation of newborn screening carries numerous scientific, clinical, ethical, and economic challenges. Not all mutations in the GLA gene result in severe disease phenotypes, e.g., female heterozygotes, late-onset variants, or VUSs may present minimal or no clinical manifestations for many years [39]. Consequently, the number of confirmed and treatment-requiring cases may remain low compared to the high cost of screening the entire newborn population. One of the most serious challenges of NBS is its diagnostic and interpretative limitations. Even though reliable and proven methods such as validated assays for α-galactosidase A activity in dried blood spots (DBSs) for screening are available, NBS cannot accurately distinguish classic from later-onset forms. The presence of variable expression and uncertain clinical relevance raise questions about the overall justification for population-wide NBS implementation. It is also important to note that enzyme-based assays fail to identify a significant proportion of female heterozygotes due to random X-chromosome inactivation, resulting in normal or borderline enzyme activity levels. To enhance the specificity of these results, second-tier confirmatory testing (e.g., lyso-Gb3 quantification) and GLA gene sequencing are added, which further increases the technical and financial burden of NBS. Large-scale testing also carries the risk of identifying benign variants or VUSs that necessitate further evaluation: confirmatory testing, clinical follow-up, and, often, long-term surveillance to determine their eventual clinical relevance. It can also reveal a certain portion of false-positive results. This not only imposes additional financial and logistical burdens on the healthcare system but, above all, causes unnecessary distress for families. The diagnosis of a potential genetic disorder in a newborn may have an enormous psychological and social impact on the child and its family and may contribute to the emergence of “vulnerable child” [46] dynamics or the perception of “patients in waiting” [47]. A positive screening result in a newborn can provoke anxiety or even stigmatization, even though clinical manifestations may be minimal or appear only in adulthood [48]. Families may experience a “medicalization” of childhood, in which an otherwise healthy child is perceived as ill or vulnerable. A Japanese study based on maternal reports highlighted the profound emotional distress and a sense of helplessness associated with prolonged uncertainty and medicalization during the asymptomatic period, particularly among mothers, who are often heterozygous carriers and primary caregivers [49]. This proves the need for genetic and psychological counseling as an integral component of NBS. Even when the diagnosis is confirmed, substantial doubts regarding the initiation of treatment in asymptomatic or mildly affected individuals remain. Given that treatment is costly and burdensome, its appropriate use is mandatory, while no universally accepted guidelines regarding the optimal timing of treatment initiation in asymptomatic individuals exist. Inappropriate or premature treatment could lead to unnecessary exposure, increased healthcare costs, and treatment fatigue among families. Creating standardized treatment algorithms is essential for the accurate use of available therapies.

The introduction of NBS for Fabry disease raises complex legal and ethical questions that remain insufficiently addressed. For example, screening newborns for disease that may or may not manifest until adulthood weighs the psychological burden of, on the other hand, whether the healthcare provider or family members can question patients’ “right not to know”. Issues of informed consent for genetic testing, the protection of genetic privacy, long-term data storage policies, and the broader ethical implications of early genetic diagnosis are still new subjects and many jurisdictions still lack clear legal frameworks.

Finally, economic and logistical considerations play a substantial role. Both diagnostic procedures and treatment are costly and burdensome; therefore, asymptomatic carriers, later-onset patients, and VUSs add costs without clear evidence of improved health outcomes. Furthermore, the implementation of NBS programs will raise the need for specialized centers, trained personnel, and data management systems.

At the moment, we do not have an ideal NBS method and only long-term observations could show the impact of early diagnosis. To justify the inclusion of Fabry disease screening in a public health program, certain prerequisites must be met. Firstly, the screening assays (enzymatic, genetic, or biomarker-based) must demonstrate a high sensitivity and specificity. Secondly, the detection of a mutation must be linked to a clearly defined management pathway, including standardized care protocols, monitoring strategies, and access to therapy. Finally, a comprehensive system must be established to provide families with information, genetic counseling, and psychosocial as well as logistical support. Long-term follow-up will be necessary in order to assess the health–economic impact of NBS and validate its potential as a public health measure for FD. However, in several studies considering the patients’ perspective, NBS, despite its limitations, was generally approved [50,51,52], as it offers a chance not only for a proper diagnosis and timely treatment but also to alter the decision-making process in important matters such as parenthood. About 42.6% of participants with FD said their lives would have been more satisfactory had they been diagnosed as newborns, as it would have eliminated diagnostic odysseys, and they were more concerned about the matter of health insurance than the possible influence on the children’s autonomy. Continued international collaboration, the unification of screening protocols, and the integration of psychosocial and ethical frameworks will be essential for maximizing the benefits of NBS while minimizing its unintended harms. Table 2 sums up the benefits and challenges of NBS.

## 7. Conclusions and Future Directions

Fabry disease is a rare genetic disorder which, untreated, leads to irreversible organ damage and premature death. The proper diagnosis is very often delayed or even missed, especially in females. The primary objective of instigating newborn screening for Fabry disease is the early identification of infants with the classic phenotype and the prompt initiation of treatment. Although numerous pilot screening programs have been conducted worldwide, NBS for Fabry disease has not yet been incorporated into the standard newborn care in any country. Beyond the evident benefits associated with early detection, NBS introduces new clinical challenges, including the interpretation of variants of uncertain significance, biomarker validation, the management of asymptomatic individuals, and decisions regarding treatment initiation. It also raises a broad spectrum of ethical issues and economic burden. Furthermore, NBS programs should serve as an important source of information and facilitate the development of regional variant databases, to characterize the controversial variants and provide clarification on the genotype–phenotype correlations. NBS could also serve as a starting point for the development of national Fabry registries. Long-term observational studies are essential for establishing standardized diagnostic protocols and evidence-based therapeutic guidelines for individuals with Fabry disease.

## Figures and Tables

**Table 1:** Diagnostic methods used in newborn screening (NBS) in Fabry disease.

Method	Biological Sample	Advantages	Limitations
Fluorometric α-galactosidase A (α-Gal A) activity assay	Dried blood spot (DBS); enzymatic assessment of α-Gal A activity	Rapid, cost-effective, suitable for high-throughput screening	False-positive results, especially in heterozygous females; requires confirmatory testing
Tandem mass spectrometry (MS/MS) α-Gal A activity assay	DBS: precise α-Gal A activity by MS/MS	High sensitivity and specificity; enables multiplex testing for other lysosomal enzymes	Requires specialized equipment and technical expertise
Biomarker quantification (lyso-Gb3)	DBS or plasma; measurement of lyso-Gb3 by (LC-MS/MS)	Correlates with disease severity; useful for confirmation and follow-up	Reduced sensitivity in females and late-onset variants
Molecular genetic analysis of the GLA gene	DBS or whole-blood- DNA sequencing	Provides definitive diagnosis and variant classification	Expensive; may detect variants of uncertain significance (VUSs); requires expert genetic interpretation
Combined enzymatic and molecular testing	DBS; enzyme activity measurement and GLA gene analysis	Minimizes false-positive results; improves diagnostic accuracy	Higher cost and longer turnaround time

**Table 2:** Benefits and challenges of newborn screening (NBS) for Fabry disease.

Category	Benefits	Challenges
Early detection	Identification of affected individuals before symptom occurs; allows genetic counseling and early intervention	Detection of late-onset variants/VUSs/benign variants/false positive
Treatment outcomes	Timely initiation of ERT or chaperone therapy, may prevent irreversible organ damage	Costly and burdensome therapy with limited evidence in long-term outcomes when started in asymptomatic newborns
Family cascade testing	Identification of other affected relatives through genetic tracing	May raise ethical concerns regarding psychological impact on entire family
Public health benefits	Increased awareness and understanding of Fabry disease; creating data registry for epidemiological insights	Requires substantial resources, trained personnel, and standardized follow-up protocols
Diagnostic accuracy	Screening tests are increasingly precise with combined enzymatic and molecular assays	Both false negatives and positives in heterozygous females; interpretation of VUSs remains problematic
Cost-effectiveness	?	Depends on prevalence and treatment cost
Ethical and psychosocial aspects	Supports life important decisions: reproductive decisions, lifestyle, disease management	Ethical controversies about screening asymptomatic individuals for late-onset diseases: “vulnerable children” and “patients in waiting”

?: It means that cost-effectiveness of NBS is questionable and yet to be proved.

## Data Availability

No new data were created or analyzed in this study. Data sharing is not applicable to this article.

## Abbreviations

The following abbreviations are used in this manuscript:

DBSs	dried blood spots
DMF	digital microfluidics
ERT	enzyme replacement therapy
FOS	Fabry Outcome Survey
Gb3	globotriaosylceramide
LOS	loss-of-function
lyso-Gb3	globotriaosylsphingosine
MS	mass spectrometry
NBS	newborn screening
VUSs	variants of uncertain significance
α-Gal A	α-galactosidase A
